# Regional disparities and seasonal differences in climate risk to rice labour

**DOI:** 10.1088/1748-9326/ac3288

**Published:** 2021-11-15

**Authors:** Charles Simpson, J Scott Hosking, Dann Mitchell, Richard A Betts, Emily Shuckburgh

**Affiliations:** 1 British Antarctic Survey, High Cross, Cambridge CB3 0ET, United Kingdom; 2 Institute for Environmental Design and Engineering, University College London, Central House, 14 Upper Woburn Place, London WC1H 0NN, United Kingdom; 3 The Alan Turing Institute, 96 Euston Rd, Somers Town, London NW1 2DB, United Kingdom; 4 University of Bristol, University Road, Clifton, Bristol BS8 1SS, United Kingdom; 5 Met Office Hadley Centre, FitzRoy Road, Exeter EX1 3PB, United Kingdom; 6 University of Exeter Global Systems Institute, Laver Building, North Park Road, Exeter EX4 4QE, United Kingdom; 7 Department of Computer Science and Technology, University of Cambridge, JJ Thomson Ave, Cambridge CB3 0FD, United Kingdom

**Keywords:** seasonal, climate change, agriculture, heat stress

## Abstract

The 880 million agricultural workers of the world are especially vulnerable to increasing heat stress due to climate change, affecting the health of individuals and reducing labour productivity. In this study, we focus on rice harvests across Asia and estimate the future impact on labour productivity by considering changes in climate at the time of the annual harvest. During these specific times of the year, heat stress is often high compared to the rest of the year. Examining climate simulations of the Coupled Model Intercomparison Project 6 (CMIP6), we identified that labour productivity metrics for the rice harvest, based on local wet-bulb globe temperature, are strongly correlated with global mean near-surface air temperature in the long term (*p* ≪ 0.01, *R* ^2^ > 0.98 in all models). Limiting global warming to 1.5 °C rather than 2.0 °C prevents a clear reduction in labour capacity of 1% across all Asia and 2% across Southeast Asia, affecting the livelihoods of around 100 million people. Due to differences in mechanization between and within countries, we find that rice labour is especially vulnerable in Indonesia, the Philippines, Bangladesh, and the Indian states of West Bengal and Kerala. Our results highlight the regional disparities and importance in considering seasonal differences in the estimation of the effect of climate change on labour productivity and occupational heat-stress.

## 1. Introduction

Agricultural workers are especially vulnerable to hot and humid weather, which impacts health and productivity. There are 880 million agricultural workers worldwide (2019 estimate) [1], the majority in low-income countries (LIC) and lower-middle income countries (LMIC). Field studies have demonstrated the presence of heat strain and related health issues in agricultural workers [2–4]. In some places, workers are frequently subject to temperatures in the range considered harmful. To cope with heat stress, workers reduce their work pace, implying a wellbeing trade-off between productivity and thermal comfort. People in the tropics, and outdoor workers especially, will be exposed more frequently to hot and humid conditions in the future due to climate change [5–7].

Ninety percent of global rice production is in Asia, equivalent to 630 million tonnes per year (in 2010–2012) [8, 9]. The majority of rice cropland is tropical or subtropical (57% between 23.45 deg N and S, 95% between 35 deg N and S). In the Asia and Pacific region, agriculture employs more than 580 million people (2019 estimate) [1], and rice production comprises 20% of total crop gross production value (2018 estimate) [10]. The labour of rice production is not evenly distributed through the year, and in some locations rice is harvested during the hottest months of the year [4]: this demonstrates that assessments of future impacts on labour productivity should incorporate the seasonality of agricultural labour.

In this article, we estimate the labour productivity effects of climate change for rice harvesting specifically, based on data from global climate models, weather reanalysis, and a database of rice production. Other studies have made estimates of the labour productivity effects of climate change in a more general scope [6, 11–13]; we examine some of the assumptions used in these studies. We identify the distribution of labour through the year as an important assumption in relation to agricultural production. We also note the importance of mechanization, the potential for adaptation to higher heat conditions, and the difficulty of accounting for these factors.

## 2. Methods

The section 2 is organised as follows. Section 2.1 describes the climate data used in this study, especially global climate model outputs. Section 2.2 describes the calculation of heat-stress metrics from the climate data. Section 2.3 describes the rice harvest data. Section 2.4 explains the statistical experiments and comparisons that were made.

### 2.1. Climate data

In this study we analyse data from the Coupled Model Intercomparison Project, Phase 6 (CMIP6) [14], comprised of global climate models that have been run in a shared experimental configuration. The model outputs used here are daily mean and maximum temperature, specific humidity, and surface-level air-pressure. All 14 CMIP6 models for which appropriate data were present in the Centre for Environmental Data Analysis (CEDA) archive^8^ 8
http://archive.ceda.acuk/.
 were included. CMIP historical runs and ScenarioMIP future pathways were processed. ScenarioMIP runs simulate the future climate given assumptions about future emissions (especially of greenhouse gases) and development pathways called shared socioeconomic pathways (SSPs) [15]. So that models with multiple ensemble members were not given additional statistical weight, a single ensemble member was used for each model, selected in numerical order from what was available in the archive. A table of model runs used, with data citations, is included in the supplementary material available online at stacks.iop.org/ERL/16/124004/mmedia (supplementary tables 2 and 3). Emissions trajectories in ScenarioMIP are derived from socioeconomic assumptions; we only made use of the atmospheric variables output from the climate models and did not make any direct use of the socioeconomic data. The Climatic Research Unit gridded Time Series (CRUTS) 4.03 [16, 17], and the European Centre for Medium-Range Weather Forecasts Reanalysis 5 (ERA5) [18], were used as historical observational datasets.

Daily frequency CMIP6 data were used. Sub-daily data for the variables of interest were available for only a small number of models. Daytime temperature variation was estimated by assuming the temperature is close to the daily mean temperature, daily max temperature, and the mid-point of the two, for 4 h each, following Kjellstrom *et al* [6]. Comparing the result of this calculation using 3-hourly and daily CMIP6 data, we found that this is a reasonable approximation. Daily ERA5 data were used to maintain consistency with the CMIP6 data. CRUTS 4.03 is monthly and is only included as a crosscheck.

### 2.2. Heat stress calculations

Wet-bulb globe temperature (WBGT) is a heat-stress metric defined by ISO 7243 and widely used for assessing hazards due to hot conditions [19–21]. WBGT is intended to combine the factors that affect the human experience of heat: namely air temperature, radiant temperature, humidity, and air velocity [20]. WBGT is designed to be measured directly using specialised equipment; in practice, statistical and empirical formulae for estimating it from standard meteorological variables must be used in the climate context. We assumed that work occurs in the shade, so that air temperature approximates black-globe temperature. Furthermore, cloud cover is one of the most uncertain aspects of global climate models [22]. In the supplementary material (supplementary section 2.1), we explore the effect of excluding radiation. Although this leads to underestimation of heat stress in some conditions, we find that long-term trends in WBGT are determined mainly by changes in air temperature. Furthermore, this assumption is used in other related studies [6, 11, 12].

WBGT was calculated as ${\text{WBGT }} = { }0.7{\text{*WBT }} + { }0.3{\text{*BGT}}$, where WBT is the wet-bulb temperature. WBT was calculated from air temperature, specific humidity, and air pressure using open-source software ‘psychrolib’ [23] which implements formulae from the American Society of Heating, Refrigerating and Air-Conditioning Engineers handbook. Field measured WBT decreases with wind speed at low speed (<2 m s^−1^, a light breeze), but higher wind speeds have a lesser effect [24]. The WBT calculation used is consistent with a light breeze, and variation in wind speed is neglected.

Studies of occupational heat stress under climate change often either assume a threshold in WBGT above which a worker is at risk [25], or assume that there is a simple relationship between WBGT and worker productivity. Typically, these relationships are assumed to be representative across sectors, and are based on either a regulatory or advisory standard [11], limited field study or survey data, or an ad hoc fusion of the two [6, 12, 26]. Occupational heat-stress regulations attempt to minimise harm and are often exceeded in practice. Therefore, use of regulatory standards to estimate future labour-productivity, as in Dunne *et al* [11], may not be accurate. Field data measuring the effect of heat on worker productivity are sparse and cover only a few activities. Studies of small numbers of workers are often used to estimate productivity effects on the entire human population.

Sahu *et al* [4] observed a 5% per °C WBGT decrease in the labour capacity of labourers harvesting rice between 26 °C and 32 °C WBGT. Rate of collection was measured in 124 workers in groups of 10–18, and WBGT was measured *in-situ*, at an individual location in India. For our study, we assume that this is representative of manual rice harvest labour, and adopt it as our labour impact metric. By labour impact metric, we mean the amount that labour productivity is reduced by heat stress. The impact is assumed to be linear in WBGT, although this assumption must break down at high WBGT (>35 °C). The systematic uncertainty due to these assumptions cannot be assessed without larger scale field observations. The labour impact metric is calculated as $5.14{\text{*WBGT}} - { }218$, in units of %, clipped at 0 and 100, meaning that 0% loss occurs at 23 °C and 100% loss occurs at 42.5 °C. This function comes from interpretation of the Sahu *et al* [4] study by Gosling *et al* [26]. By using a labour impact derived from a study specific to manual rice harvesting, we represent this particular activity more accurately than the assumptions used for the economy in general in other studies (e.g. [6, 11, 12, 25–27]).

To facilitate comparison to other studies, results were also calculated using different assumptions about the relationship between WBGT and labour productivity: the assumptions used for agricultural labour in Dunne *et al* [11], and in Orlov *et al* [27]. Our calculations are consistent with these other studies above. In the supplementary material (supplementary section 2.2), equations for each of these and the effect of varying the assumptions are shown.

### 2.3. Rice data

**Figure 1.**
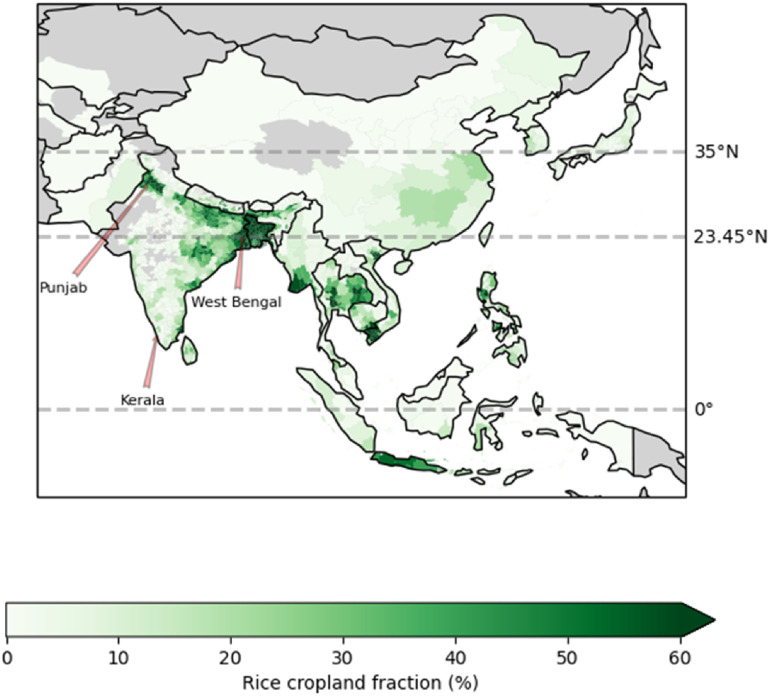
Geographical distribution of rice production across Asia (green shading). Sub-national locations mentioned in the article are labelled. Data sourced from RiceAtlas.

**Figure 2.**
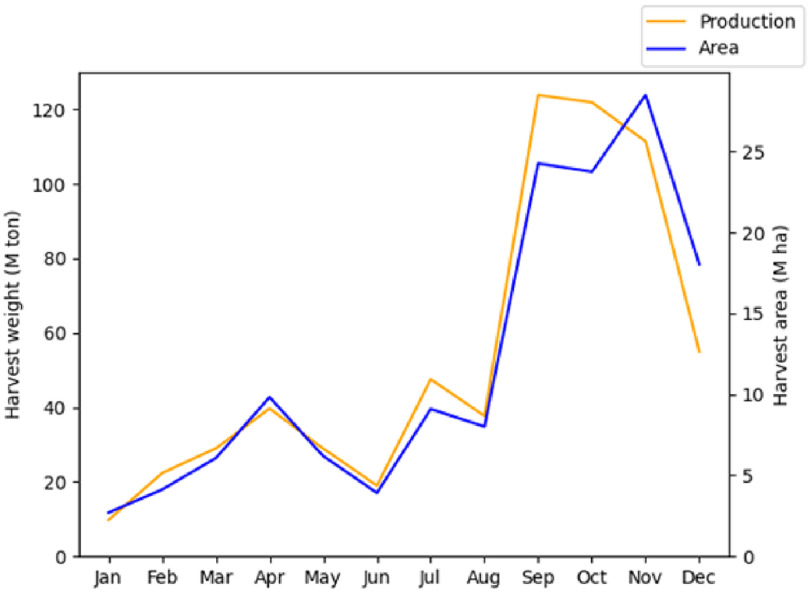
Rice harvest weight and area in Asia, plotted against month. Data from RiceAtlas.

**Figure 3.**
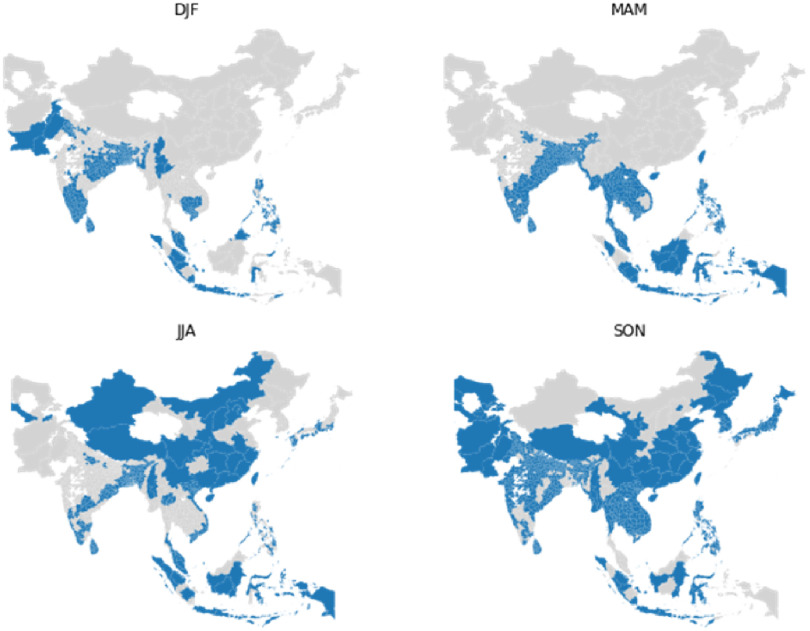
Locations of a rice harvests by season. Areas with a rice harvest in a given season are shown in blue. Data from RiceAtlas.

The RiceAtlas dataset [8, 9] provides detailed data on rice production, broken down into location entities with an average area of 5000 km^2^. The distribution of rice cropland in Asia is shown in figure 1. Information such as yield, harvested area, planting and harvesting dates are included, and many entities have multiple yearly harvests. The data are representative of the years 2010–2012. We used this to identify harvest dates. Figure 2 shows the distribution of rice harvests through the year across the whole of Asia according to RiceAtlas. Figure 3 shows where harvests occur in each calendar season. Harvests peak in September-November, but there is a large amount of spatial variation. For a more complete illustration of the timing of harvest seasons in the RiceAtlas dataset, we refer to the paper describing that dataset.

Location entities in RiceAtlas vary greatly in size, and many are much smaller than the grid spacing of the climate models considered. Where a location geometry enclosed multiple climate-model grid-cells, the mean was taken of the enclosed cells; otherwise, the land grid-point closest to the centroid of the location was used. We used data only for countries in Asia; see table 1.

**Table 1.** Rice production exposed to hazard gradient above the 50th percentile by country, and Asia total. Countries with less than 1 million tonnes of annual rice production are not included. Rice production from RiceAtlas. We define hazard gradient as the gradient between GSAT and local labour impact.

Country	Production above 50th percentile of hazard gradient (%)	Total production (million tonnes)	Production above 50th percentile of hazard gradient (million tonnes)
Asia	50	646	323
Indonesia	100	67	67
India	36	151	55
China	27	200	55
Vietnam	83	42	35
Thailand	77	36	28
Bangladesh	61	45	27
Myanmar	71	32	23
Philippines	94	17	16
Cambodia	75	9	7
Sri Lanka	100	4	4
Malaysia	100	3	3
Japan	29	8	2
Taiwan	70	2	1
Laos	25	3	1
South Korea	0	6	0
Nepal	0	5	0
North Korea	0	2	0
Pakistan	0	9	0
Iran	0	3	0

### 2.4. Statistical experiments and comparisons

For each location and harvest season, climate data (ERA5 and CRUTS 4.03, and CMIP6 outputs) were selected using the harvest dates and spatial geometry provided by RiceAtlas, and WBGT calculated. Only climate data during the peak month of each harvest were selected. Then, the labour impact metric was calculated from WBGT. Independently for each climate model, and for each location and season, a linear trend was fitted between the 20 year means of the change in the global mean near-surface air temperature (GSAT) and the labour productivity metric. Twenty-year periods were defined between 1850 and 2009 inclusive for the historical runs, 2020–2099 inclusive for the SSP runs. As the labour productivity function was clipped at zero, the fitted function was too. We assumed that 1 °C of GSAT warming relative to 1850–1900 was representative of the present (2020) [28], and changes in both variables are relative to the present. Data from historical model runs and different SSPs were included together in a single fit, so that a range of GSAT values (up to 4 °C relative to 1850–1900) were included; but each model, and harvest season and location was fit independently. We defined the gradient of this fit as the ‘hazard gradient’, the purpose of which is to summarise the relationship between changes in GSAT and local changes in labour productivity via WBGT, to explore the spatial patterns of change. The hazard gradient means the amount by which the labour impact increases on average for 1 °C of global warming. This effectively collapses variation in time, the climate sensitivity of each model, and emissions scenario into a single dimension. The two-sided inverse Student’s *t*-distribution was used to determine whether the hazard gradient in each harvest season and location was significant.

The multi-model mean of the hazard gradient was calculated for each harvest season and location. The labour impact was also averaged over all locations, weighted by total rice production, to characterise the regional situation.

Statistical tests were applied to the results. In order to test if there was a significant change in the historical period, linear regression was used on the time-series of the labour impact metric calculated from ERA5 and CRU-TS, and the two-sided inverse Student’s *t*-distribution used to determine if the gradient was significantly different from zero. To test if there is a significant difference between different warming scenarios, the distributions of results across climate models at 1.5 °C, 2.0 °C, and 3.0 °C of global warming were compared using the Kolmogorov–Smirnov (KS) test and Student’s *t* test.

## 3. Results

### 3.1. Regional results

**Figure 4.**
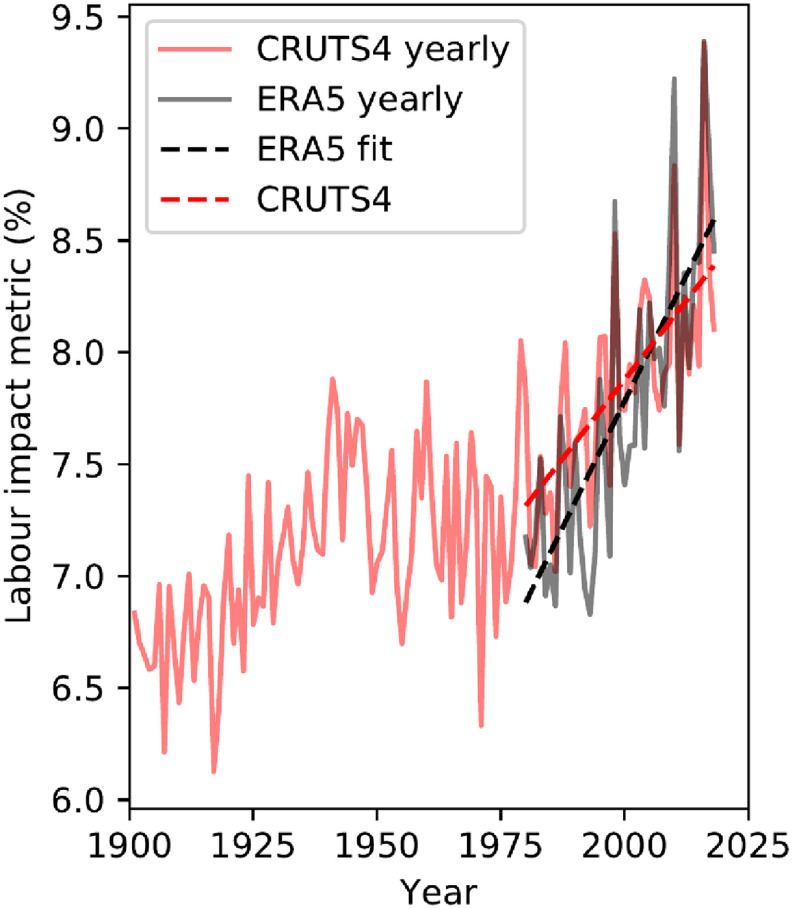
Annual Asia-wide mean labour impact, weighted by total rice production, calculated from CRU-TS 4.03 (red lines) and ERA5 (black line), with trend lines (dashed) shown for the period 1980–2018.

Figure 4 shows the Asia-wide average of the heat impact on rice harvest labour, calculated from the historical observational and reanalysis datasets, weighted by total rice production in each harvest season and location. There is a statistically significant increase (*p* ≪ 0.01) over the full observational period (1900–2018) of CRU-TS, and in both datasets for the common observational period (1980–2018): the heat hazard associated with the rice harvest has already increased.

**Figure 5.**
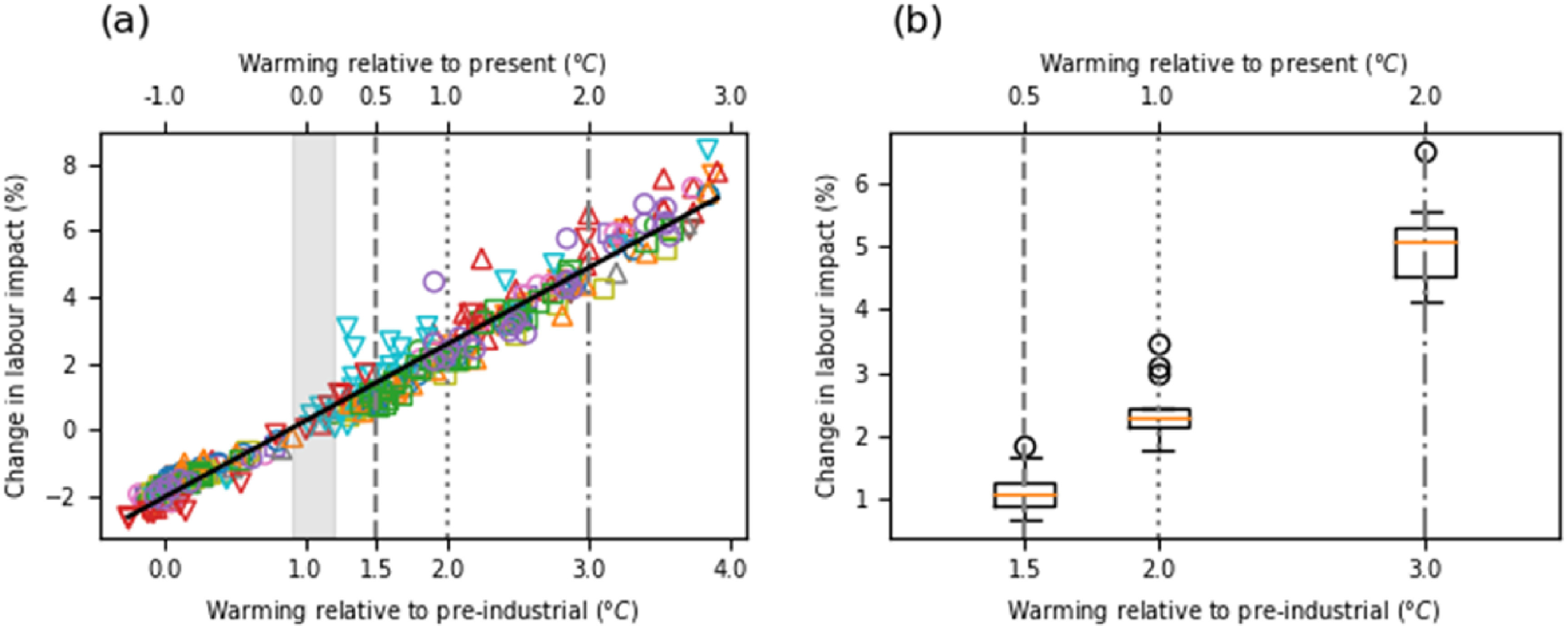
Relationship between rice labour impact and GSAT change in the 14 analysed CMIP6 climate models; Changes are relative to 1.0 C of warming relative to 1850–1900, which is assumed to represent the present. Three levels of warming relative to 1850–1900 and relative to the present are shown for context. The present estimated range of warming is shown by the grey shading. (a) Change in labour impacts against GSAT for 20 year periods; each shape/colour corresponds to a different model The black trend line is illustrative only: fit statistics mentioned in the text are based on fitting each climate model individually. (b) Change in labour impacts at three levels of warming, with box plot to show climate model spread. Points are linearly interpolated to the three levels of warming. Boxes show 1st (Q1) and 3rd quartile (Q3); orange line at the median; lower whiskers at lowest point above Q1–1.5*(Q3–Q1), upper whiskers highest point below Q1 + 1.5*(Q3–Q1); circles are points outside the whisker range. We define hazard gradient as the gradient between GSAT and local labour impact.

Figure 5 shows heat impact on Asia-wide rice harvest labour plotted against GSAT warming across various climate models. Changes are relative to the present, assumed to mean 1 °C of warming over 1850–1900. The rice harvest labour impact is weighted by total rice production. In figure 5(a), each point is a 20 year mean in a single model simulation. Despite the different biases and climate sensitivities of the models, they each show a linear relationship between GSAT and the rice harvest labour impact. All models have a hazard gradient 2.0–2.7% °C^−1^, *p* ≪ 0.01, *R* ^2^ > 0.98. The multi-model mean (standard deviation) of the hazard gradient is 2.3 (0.2)% °C^−1^. A single trend line is shown in figure 5(a) for illustration only.

In figure 5(b), the Asia-wide labour impact is linearly interpolated to 1.5, 2.0 and 3.0 °C of warming (relative to 1850–1900) individually for each model, and results are shown as a multi-model boxplot. There is a statistically significant difference between the 1.0 °C and 1.5 °C of warming with multi-model mean (standard deviation) 1.0 (0.3)%; between 1.5 °C and 2.0 °C of 1.3 (0.2)%; as well as between 2.0 °C and 3.0 °C of 2.6 (0.4)%. KS test and *t* test *p*-values on the multi-model distributions are small (≪0.001) for each case.

As the Asia-wide analysis is weighted according to total rice production in each location and season, the Asia-wide results should be interpreted carefully: the average is more sensitive to locations with more intensive cropping. However, the breakdown by location shows that the trends are general, and not skewed by a single area with high cropping intensity. The correlation coefficient of the hazard-gradient between models and within each harvest season and location is 0.95, suggesting that there is generally high agreement between the models.

### 3.2. Local and seasonal results

**Figure 6.**
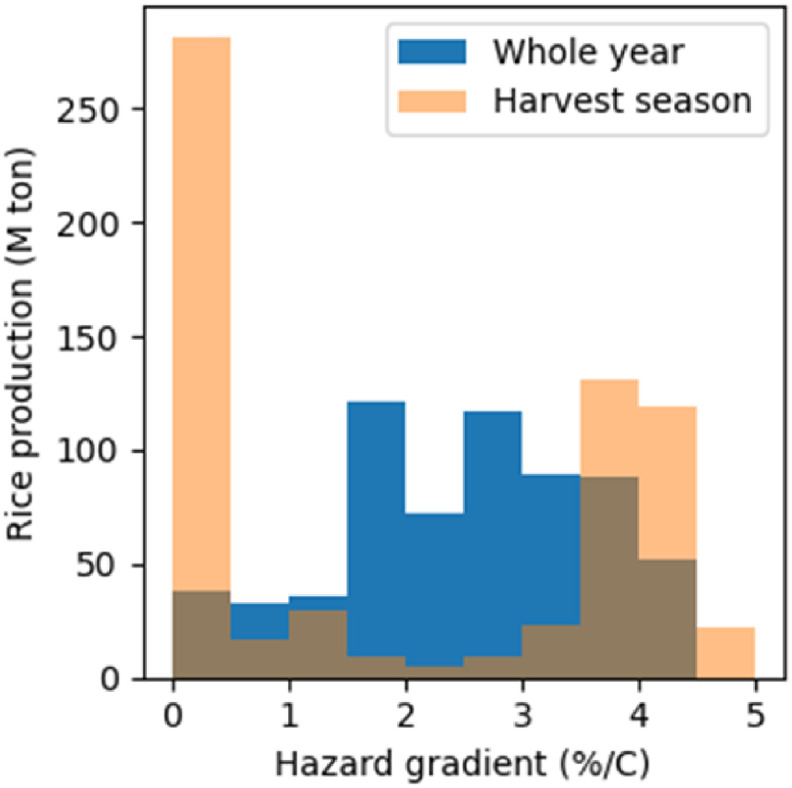
Histograms showing the distribution of hazard gradient across harvest seasons and locations. The effect of seasonal weighting assumptions on the hazard gradient is shown: the blue histogram represents the assumption that the whole year is equally weighted, while the orange histogram shows the result if only the rice harvest season is included. The histograms are weighted by the harvest weight in millions of tonnes.

Figure 6 shows the distribution of the multi-model mean hazard-gradient across harvest seasons and locations. In the blue histogram, the entire year is given equal weight in the heat stress calculation. Conversely, in the orange histogram, only the rice harvest season is considered in the heat stress calculation. Both histograms are weighted by rice production. Considering the time of year at which the rice harvest occurs, as opposed to the whole of the year, leads to some rice harvests having higher hazard-gradient, but other harvests not having a significant hazard-gradient. If the harvest season and locations with non-significant hazard-gradients are excluded, then the mean hazard-gradient is increased. The interquartile range of the distribution in orange is more than double that in blue. The two assumptions lead to completely different distributions of hazard gradient, showing the importance of including the seasonality of labour in projections of the effect of heat-stress on agricultural labour productivity.

**Figure 7.**
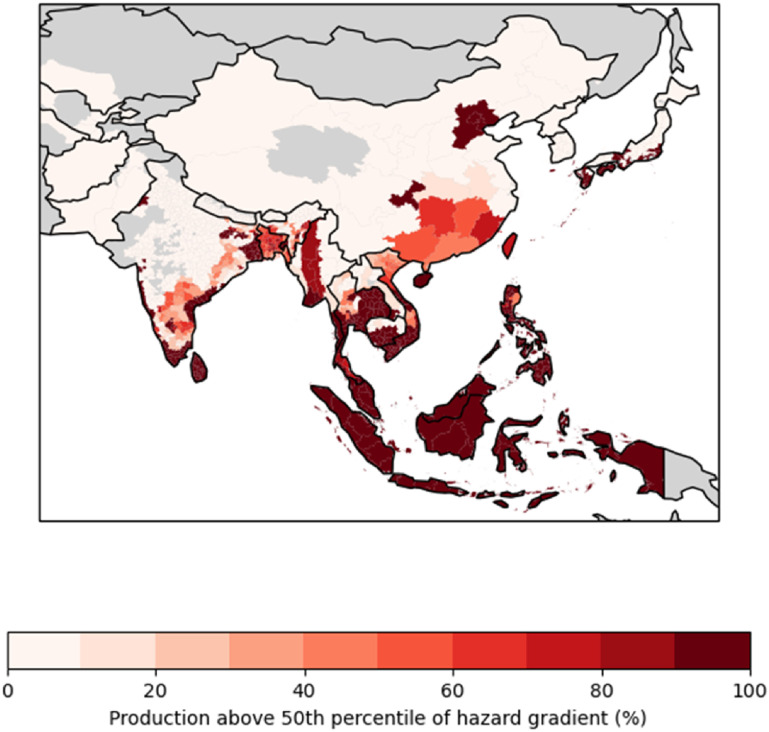
Map of Asia with shading representing proportion of rice production in harvests for which labour capacity is identified as being in the higher 50% of hazard-gradient.

The 50% of rice production with the highest hazard gradients (hazard gradient >3.05% °C^−1^) is identified as being exposed to a high hazard-gradient; the proportion of exposed production in each location is mapped in figure 7 and listed by country in table 1. We do this to identify regional differences in the estimated labour impact; we are not suggesting this is a limit to adaptation. Areas identified by this method include most of Southeast Asia, and coastal South Asia. Harvests in northern India (e.g. in Punjab) are not strongly affected.

**Figure 8.**
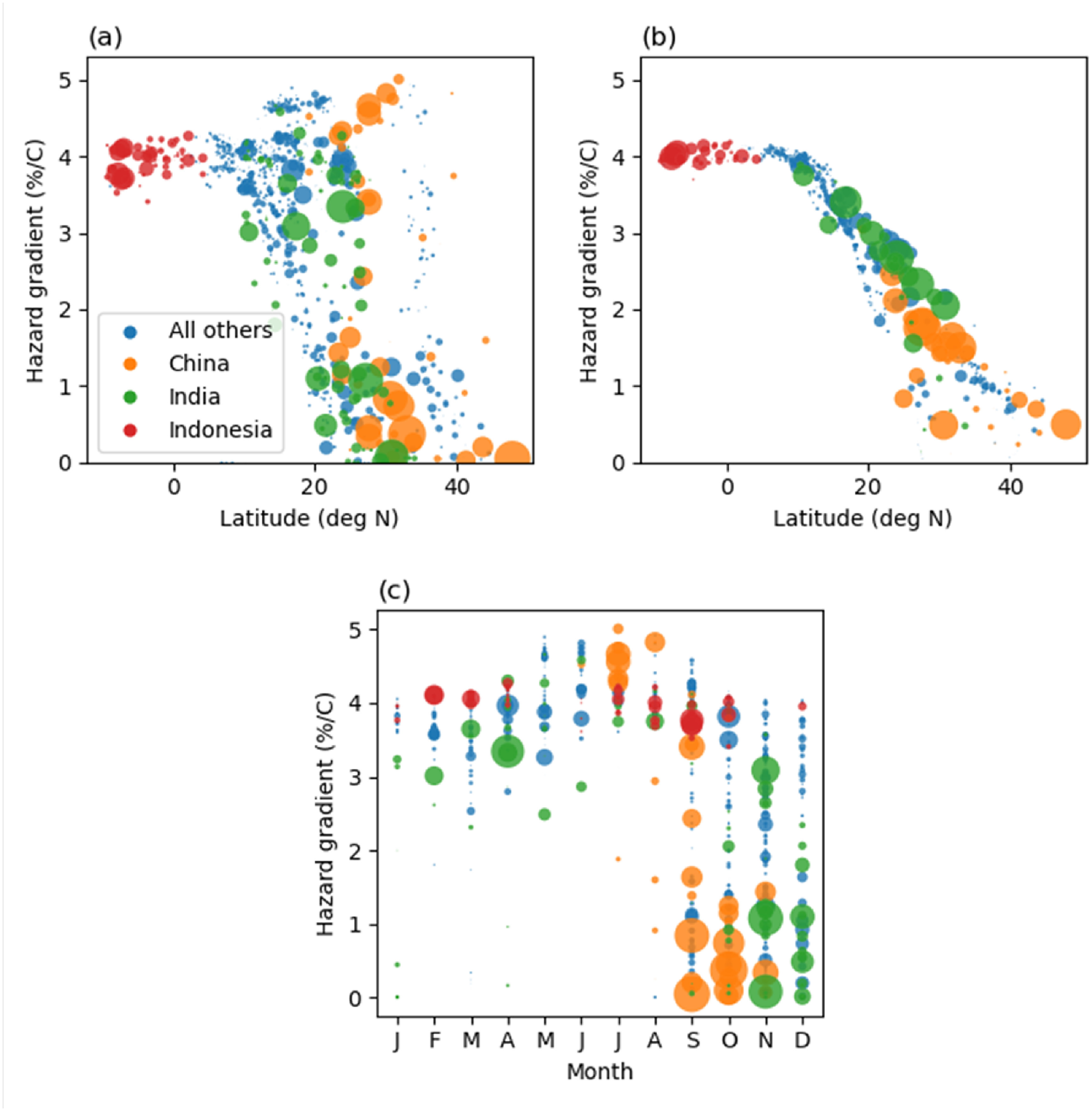
Scatter plots, with marker size proportional to total harvest weight, of hazard gradient for each location and season: (a) plotted against latitude, selecting only the peak of the rice harvest; (b) plotted against latitude, averaged over the whole year equally; (c) plotted against peak month of harvest. Colours identify points belonging to China, India, and Indonesia. Hazard gradients are multi-model means. In order to reduce the number of markers, some aggregation was performed: in both cases the mean weighted by total weight of harvested rice was taken; the results were aggregated to the 2nd level HASC geocode, independently for each month.

Figure 8 shows the hazard gradient for each harvest season and location, plotted against latitude in 6a and against the peak month of the harvest season in 6c. Figure 8(b) shows the hazard gradient against latitude assuming the full year is equally weighted. Two interacting effects explain most of the spatial variation in the results. Firstly, locations close to the equator have higher temperatures. Secondly, for locations that are further from the equator there is greater seasonal variation in temperature, so the time of year at which the harvest occurs is important. Figure 8(b) shows that if the time of year of the harvest is not considered, then the hazard gradient is mostly determined by latitude. Close to the equator, for example in Indonesia, hazard gradient is not very seasonally dependent as there is relatively little seasonal variation in temperature and harvests are spread through the year. By comparison, in China and India the time of year of the harvest season has a much stronger effect on the hazard gradient. Harvests in China occur June-November (see figure 3), with harvests occurring June-August more exposed than those occurring September-November. Figure 8(c) shows that, regionally, all large harvests that are not exposed to high hazard-gradients peak between September and December inclusive, whereas harvests July-August are more exposed.

## 4. Discussion

### 4.1. Large scale trends

Global mean surface temperature (GMST) has increased by 0.9 °C–1.2 °C relative to 1850–1900 as of 2017 [28], and if anthropogenic warming continues to follow recent trends, is projected to reach 1.5 °C between 2030 and 2052; the estimated rate of warming is 0.2 °C per decade [29]. In accordance with the Paris Agreement under the United Nations Framework Convention on Climate Change [30], most countries have committed to limit GMST warming by 2100 to 2 °C, and to make efforts to limit warming to 1.5 °C. However, at the time of writing, pledges of emissions reductions are not sufficient to meet this goal [31].

We identify in figure 4 that there is a statistically significant increase in the estimated heat impact in the historical observational dataset. Overall, the long-term averages in the estimated labour impact are highly correlated with GSAT change (figure 5). This is not surprising as the labour impact metric is linear in WBGT above a threshold, and GSAT change (i.e. global warming) dominates large-scale, long-term trends in WBGT. This relationship holds at the Asia-wide level, but also individually for most location and seasons.

### 4.2. Seasonality

Labour inputs to rice production are seasonal, with more labour occurring around planting and harvesting [32–34]. This labour cannot be delayed or displaced to other times of year, and the timeliness of these activities affects yield and quality. Averaging across the full year assumes that labour for any given activity can be exchanged with labour at a different time of year, which is clearly not the case for planting and harvesting. In this study, we have shown the importance of considering the seasonality of agricultural labour when estimating the effect of heat-stress on labour productivity. We have focussed only on harvesting, and only on rice. However, most crops are seasonal, and therefore have a seasonal distribution of labour; this should be taken into account in estimates of the effect of heat stress on labour. Other studies into the effect of climate change on labour productivity, even those focussed on agricultural labour, do not take this into account [6, 12, 13, 27].

In some cases, for example wheat in Punjab, rice is multi-cropped with another crop that is not covered in this study, but for which harvest labour may be more exposed. Studies have suggested that changes to planting and harvesting dates [35], and crop choice [36], could be used to adapt to climate change and mitigate negative effects on yields. Changes to planting and harvesting dates, to maximise yield as temperature and precipitation patterns change, is a complex topic, and outside the scope of this study. Given that the world has already experienced 1 °C of warming, such adaptations may already be occurring. Changes to planting and harvesting dates could lead to workers being exposed to heat stress in locations where they currently are not, and this should be considered when studying adaptive measures.

### 4.3. Limitations

Workers may be able to reduce heat exposure by starting work early in the morning when it is cooler, but this could mean working in the dark. Reducing work hours could also reduce heat exposure but could exacerbate labour shortage. We note that the workers observed by Sahu *et al* began their work near dawn and were still exposed to hazardous heat in their first hour of work [4].

The accuracy, coverage, and granularity of the rice crop data limits our study. However, by focussing on long-term trends with high levels of multi-model agreement, we avoid the issue of climate model bias. The assumed linear effect of WBGT on labour productivity comes from a single field study and cannot be accurate as WBGT approaches known human physiological limits, but the general conclusions are unchanged by substituting other labour-impact functions from the literature (see supplementary section 2.2). Due to the resolution of climate models, results will be less reliable for locations with steep gradients in altitude, for example Nepal.

Farming practices and machinery use mean that the required amount of labour for a given harvested area or weight will vary between places [37, 38]. Differences in mechanisation between and within LIC and LMIC are not a simple function of gross domestic product, and there is substantial variation within as well as between countries. Vulnerability may be higher in parts of sub-Saharan Africa, or for other crops, where there has been less uptake of agricultural machinery [39, 40]. This is discussed further in supplementary material section 1.

El Nino is an important driver of annual temperature variation in the region, and negatively affects crops through rainfall patterns (see e.g. Selvaraju [41]). Higher global temperatures could change El Nino patterns, but this is beyond the scope of this study. We have focussed on long term trends on the scale of 20 years rather than extreme events, but WBT extremes are also closely correlated with large-scale mean temperatures within the tropics [42].

## 5. Conclusion

The rising impact of heat on rice harvest labour will be unevenly distributed, falling on poor rural workers who will be least able to adapt, and therefore contributing to widening economic inequality. Given agriculture employs hundreds of millions of people, a few-percent shift in labour capacity is equivalent to the labour of millions of people. Labour productivity decreases due to climate change will compound other environmental impacts on rice production, including declining yields as direct result of increasing temperature [43], water stress, and sea level rise [29]; contributing to the unequal loss and damage created by climate change.

We see a strong relationship between heat hazard and GSAT change in the rice harvest seasons and locations of Asia, with a high level of agreement between climate models. Historical observational data already shows a statistically significant increase in our labour impact metric. Understanding disparities in the heat-stress exposure of workers and industries around the world will be important for climate adaptation strategy, as well as estimation of loss and damage. Overall, the exposure of such a large proportion of rice agriculture, and the workers engaged therein, provides an argument for strong mitigation of climate change.

We have chosen to focus on rice harvesting, but all of the issues discussed apply to other crops: key activities have different amenability to mechanization and different work intensities, and labour is not evenly spread throughout the year. Assuming that labour is equally distributed through the year leads to underestimation of the effect of climate change on the heat stress of agricultural workers in many places. Accurate accounting for the balance of mechanization and manual labour remains difficult.

## Data and code availability statement

The data that support the findings of this study are openly available at the following URL/DOI: 10.5281/zenodo.4737471. A code demonstration can be found at 10.5281/zenodo.4746392.
